# Editorial Note: Immunomodulation Targeting Abnormal Protein Conformation Reduces Pathology in a Mouse Model of Alzheimer’s Disease

**DOI:** 10.1371/journal.pone.0297928

**Published:** 2024-01-24

**Authors:** The PLOS ONE Editors

In this article [1], the same image is reported in Fig 7G and Fig 7J, when rotated 180 degrees.

The corresponding author stated that the data were correctly reported in Fig 7G and that an error was made in preparing Fig 7J. The corresponding author also stated that an updated panel for Fig 7J cannot be provided and that none of the raw data underlying the article’s results are currently available.

An Academic Editor advised that even considering the unresolved image issue, the article’s overall results and conclusions [1] remain supported by other results reported in the article.

The *PLOS ONE* Editors issue this Editorial Note to inform readers of the unresolved image and data unavailability* issues. While the authors are unable to correct these issues, the journal stands by the article in light of the Academic Editor’s assessment. Readers should disregard Fig 7J.

*Note: The article does not currently comply in full with the PLOS Data Policy that was in place in 2010. That policy stated that data integral to an article’s findings should be provided or made freely available and that authors must comply with current best practices in data sharing, but it did not strictly require publication or deposition of all data. PLOS updated our Data Availability Policy in 2014; see the full policy for our current data sharing requirements.
